# Oral microbiome associated with lymph node metastasis in oral squamous cell carcinoma

**DOI:** 10.1038/s41598-021-02638-9

**Published:** 2021-11-30

**Authors:** Young-Gyu Eun, Jung-Woo Lee, Seung Woo Kim, Dong-Wook Hyun, Jin-Woo Bae, Young Chan Lee

**Affiliations:** 1 Department of Otolaryngology-Head and Neck Surgery, School of Medicine, Kyung Hee University, #892 Dongnamro, Gangdon-gu, Seoul, 05278 Republic of Korea; 2 Department of Oral and Maxillofacial Surgery, School of Dentistry, Kyung Hee University, Seoul, Republic of Korea; 3 Department of Otolaryngology-Head and Neck Surgery, Veterans Health Service Medical Center, Seoul, Republic of Korea; 4 Department of Biology and Department of Biomedical and Pharmaceutical Sciences, Kyung Hee University, Seoul, Republic of Korea

**Keywords:** Microbiology, Biomarkers, Oncology

## Abstract

Oral microbiota can alter cancer susceptibility and progression by modulating metabolism and inflammation. We assessed the association between the oral microbiome and lymph node (LN) metastasis in oral squamous cell carcinoma (OSCC). We collected a total of 54 saliva samples from patients with OSCC before surgery. LN metastasis was assessed based on postoperative pathological examination. We used QIIME2, linear discriminant analysis effect size (LEfSe), and PICRUSt2 methods to analyze microbial dysbiosis. A random forest classifier was used to assess whether the oral microbiome could predict LN metastasis. Among the 54 OSCC samples, 20 had LN metastasis, and 34 had no evidence of metastasis. There was a significant difference in β-diversity between the metastasis and no metastasis groups. Through LEfSe analysis, the metastasis group was enriched in the genera Prevotella, Stomatobaculum, Bifidobacterium, Peptostreptococcaceae, Shuttleworthia and Finegoldia. Pathways related to signal peptidase II were predominant in the no metastasis group. The RF model showed a modestly high accuracy for predicting metastasis. Differences in microbial community composition and functions were observed in the oral microbiome of patients with OSCC with and without LN metastasis. However, the finding that specific taxa may be associated with LN metastasis should be verified in a further prospective study.

## Introduction

Lymph node (LN) metastasis in oral squamous cell carcinoma (OSCC) is observed in 12–50% of patients, depending on the location of the primary carcinoma^1^. The presence of LN metastasis is associated with an advanced disease, reducing the overall survival (OS) rate of OSCC patients to 50%^2^. Although clinical evaluation, including computed tomography (CT), magnetic resonance imaging (MRI), ultrasonography (US), and positron emission tomography (PET)-CT, are used to evaluate LN metastasis of oral cancer before treatment, occult LN metastasis may still occur. Nevertheless, therapeutic neck dissection (ND) is performed in patients with OSCC with clinically positive LN metastasis, but controversy remains as to whether selective ND should be performed in patients without LN metastasis^1,3^. Therefore, prediction of LN metastasis in OSCC is important not only for diagnosis, but also for establishing a therapeutic strategy. In this respect, biomarkers associated with metastasis are useful tools for identifying invasion and metastasis, and monitoring treatment response in patients undergoing cancer treatment. Several histopathological parameters, gene expression signatures, and immunohistochemical markers have been reported as biomarkers for predicting LN metastasis in patients with OSCC^4–6^. However, most existing methods have limited predictive value, and surgical resection is often required to obtain precise clinical information.

With more than 700 species of microbes, the oral cavity is one of the most densely populated human microbial habitats^7^. Oral microbiota may play a role in carcinogenesis. The mechanisms by which changes in the human microbiome are involved in the carcinogenesis process include (i) induction of a chronic inflammatory response, (ii) induction of anti-apoptotic signals in cells, (iii) release of carcinogens, and (iv) regulation of anti-cancer immunity^8^. The mucosal microenvironment is a specific ecological niche. In this system, bacteria can genetically influence the epithelial cells^7^. Furthermore, the microbiome is accepted as a new component of the tumor microenvironment that impairs tumor cell metabolism by maintaining mucosal barriers or causing inflammation and producing genetic toxins and bacterial metabolites with various functions^9^. A number of microorganisms have been associated with the promotion of carcinogenesis or metastasis through manipulation of the *Wnt* signaling pathway, which is associated with the E-cadherin–catenin complex^10^. E-cadherin and related catenin complexes play an important role in cell adhesion, and misregulated E-cadherin is believed to be a key step in cancer metastasis^11^. The squamous epithelium of the oral mucosa is constantly exposed to various oral microbes, both of which communicate with each other at the cellular and molecular level^12^. Thus, there have been several studies over the past decades suggesting a link between oral cancer and oral microbes^13^. Previous studies have reported that several specific oral microbiomes, such as Streptococcus, Peptostreptococcus, Prevotella, *Porphyromonas gingivalis*, and *Capnocytophaga gingivalis*, are strongly associated with oral squamous cell cancer^14^. A recent meta-analysis showed thar *Fusobacterium* is more abundant in head and neck cancer (HNC) samples than non-HNC samples, suggesting the possible contribution of *Fusobacterium* to the carcinogenesis of OSCC^15^. In particular, *Fusobacterium nucleatum* may play an important role in tumorigenesis of oral cancer through several mechanisms^16^.

Although the role of oral microbiota in the pathogenesis of OSCC is becoming more evident, little is known about its association with the occurrence of metastasis. We hypothesized that the oral microbiome is related to metastasis in OSCC patients. Using saliva samples, we compared the abundance of microbial taxa and functional diversity between OSCC patients with and without LN metastasis.

## Results

### Baseline characteristics

Among the 54 OSCC patients, 20 had LN metastasis in the postoperative pathologic report, and 34 had no evidence of metastasis. There were no significant differences in age, history of smoking, and denture use between the two groups. The proportion of male patients and the number of patients with a higher T stage in the LNM group was higher than in the non-LNM group (Table 1).

**Table 1:** Baseline characteristics of enrolled oral squamous cell carcinoma patients.

	No LN metastasis group	LN metastasis group	*p* Value
Sample number	34	20	
Age	64.0 ± 11.7	69.0 ± 11.2	0.132
**Sex**			0.004
Male	21 (61.8%)	20 (100.0%)	
Female	13 (38.2%)	0 ( 0.0%)	
Current smoker	14 (41.1%)	11 (55.0%)	0.078
Denture use	0 (0%)	3(15.0%)	0.092
**T-stage**			0.003
I	12 (35.3%)	0 ( 0.0%)	
II	12 (35.3%)	6 (30.0%)	
III	3 ( 8.8%)	8 (40.0%)	
IV	7 (20.6%)	6 (30.0%)	

LN: lymph node.

### Diversity analysis

**Figure 1:**
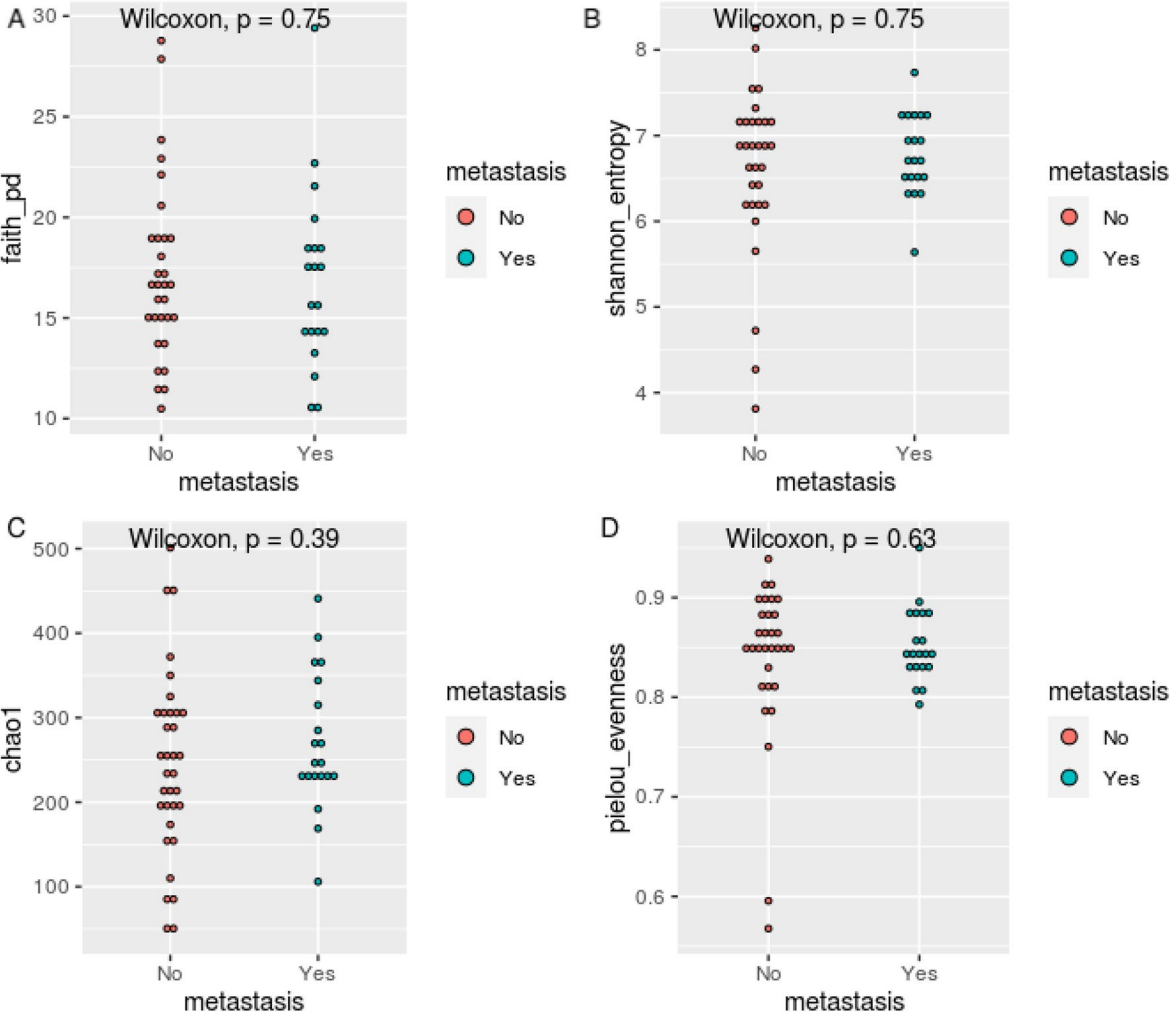
α-diversity in oral squamous cell carcinoma patients with metastasis and no metastasis. The α-diversity in terms of faith_pd (**A**), shannon_entrophy (**B**), chao1, (**C**) and pielou_evenness (**D**) are compared.

**Figure 2:**
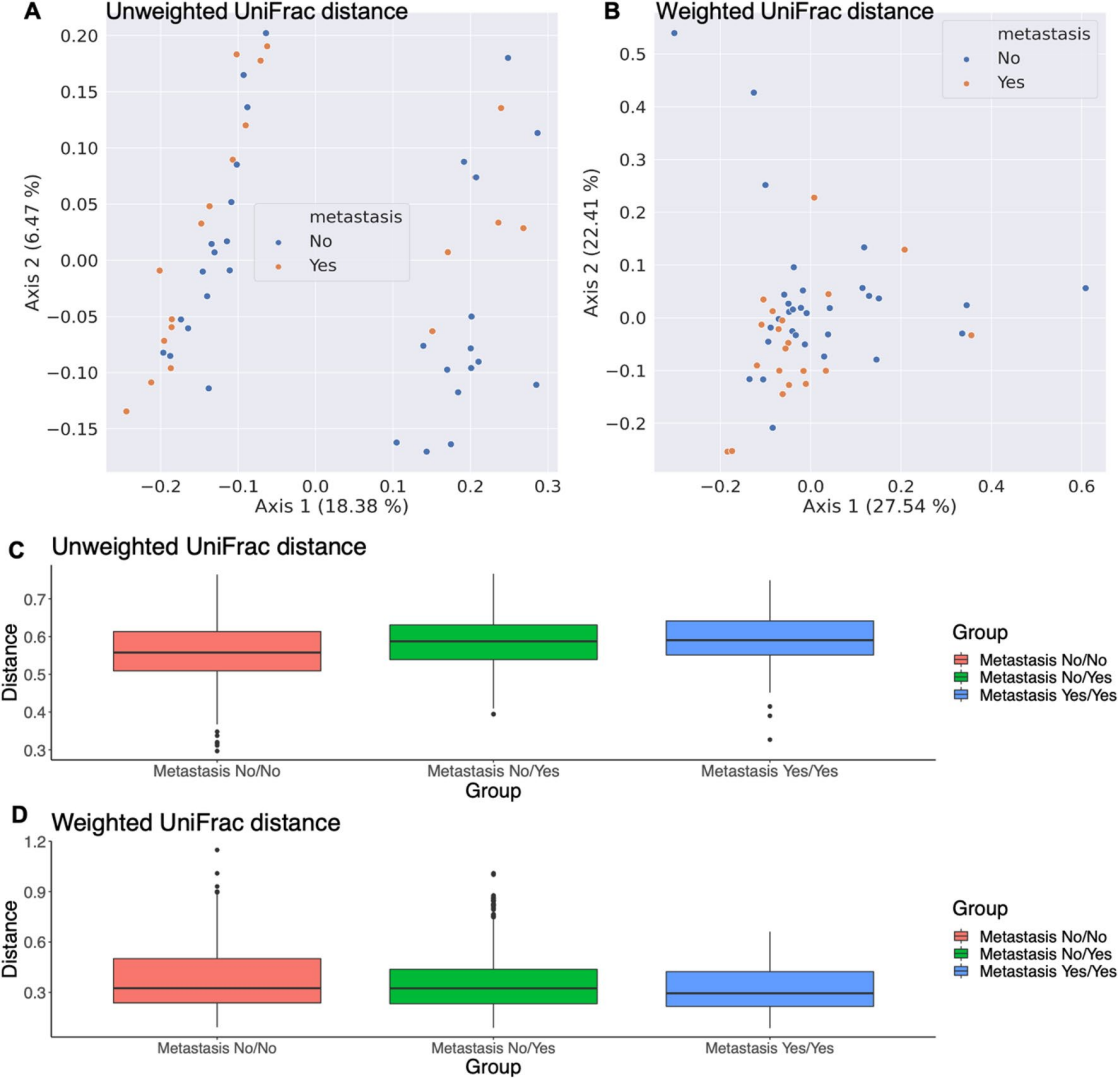
β-diversity in oral squamous cell carcinoma patients with metastasis and no metastasis. Principal coordinated analysis of unweighted (**A**) and weighted (**B**) unifrac distance of oral microbiota. Each point represents individual sample. Average unweighted (**C**) and weighted (**D**) unifrac distance representing intragroup and intergroup within oral microbiota. Permutational multivariate analysis of variance was performed with the unweighted unifrac distance index (Permanova *p* = 0.031) and weighted unifrac distance index (Permanova *p* = 0.069). Metastasis yes/yes in X axis means the sequence distance between one sample in the yes group and another sample in the yes group.

Alpha and beta diversity indices calculated on the ASV level are shown in Figs. 1 and 2, respectively. In terms of α-diversity, no significant difference was found between metastasis and no metastasis group according to faith_pd index (*p* = 0.75), Shannon entropy (*p* = 0.75), chao1index (*p* = 0.39) or the pielou evenness index (*p* = 0.63). (Fig. 1) For β-diversity, we calculated average unweighted and weighted unifrac distance representing intragroup and intergroup within oral microbiota. Unweighted unifrac measures the fraction of unique branch length and weighted unifrac considers abundance. Principal coordinated analysis (PCoA) plots showed distinct clustering between metastasis and no metastasis group in unweighted unifrac distance, but not in weighted unifrac distance. We observed significant differences in the unweighted unifrac distance index between the two groups (Permanova p = 0.031) (Fig. 2A–D).

### Differential oral microbiota and functional analysis

**Figure 3:**
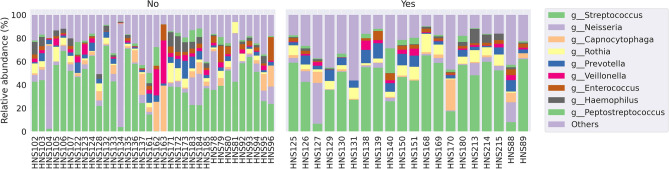
The relative abundance of oral bacterial communities at the genus level between the two groups.

Oral bacterial communities were explored at genus levels in the patient samples (Supplemental Fig. 1). Frequent genera were Streptococcus, Neisseria, Capnocytophaga, Rothia, Prevotella, Veillonella, Enterococcus, Haemophilus and Peptostreptococcus. Figure 3 shows the comparison in relative abundance of oral bacterial communities at the genus level between the two groups.

**Figure 4:**
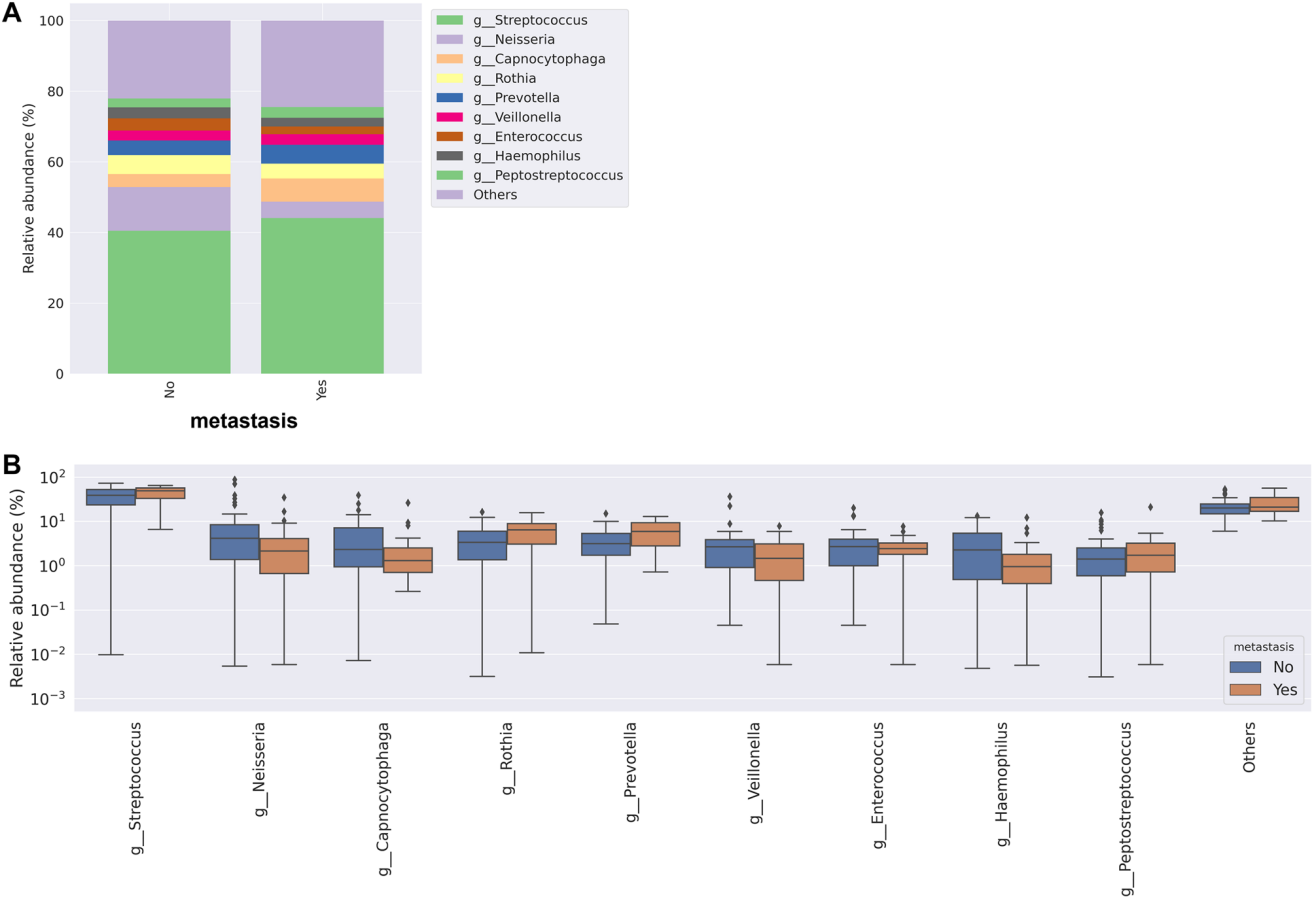
Relative abundance of oral bacterial communities at the genus level between the two groups. (**A**) Mean relative abundance of 10 taxa between the two groups (**B**).

When comparing the relative abundance at the genus level between the two groups, it was observed that Streptococcus, Rothia, Prevotella and Peptostreptococcus increased, but Neisseria and Haemophilus decreased in the metastasis group (Fig. 4).

**Figure 5:**
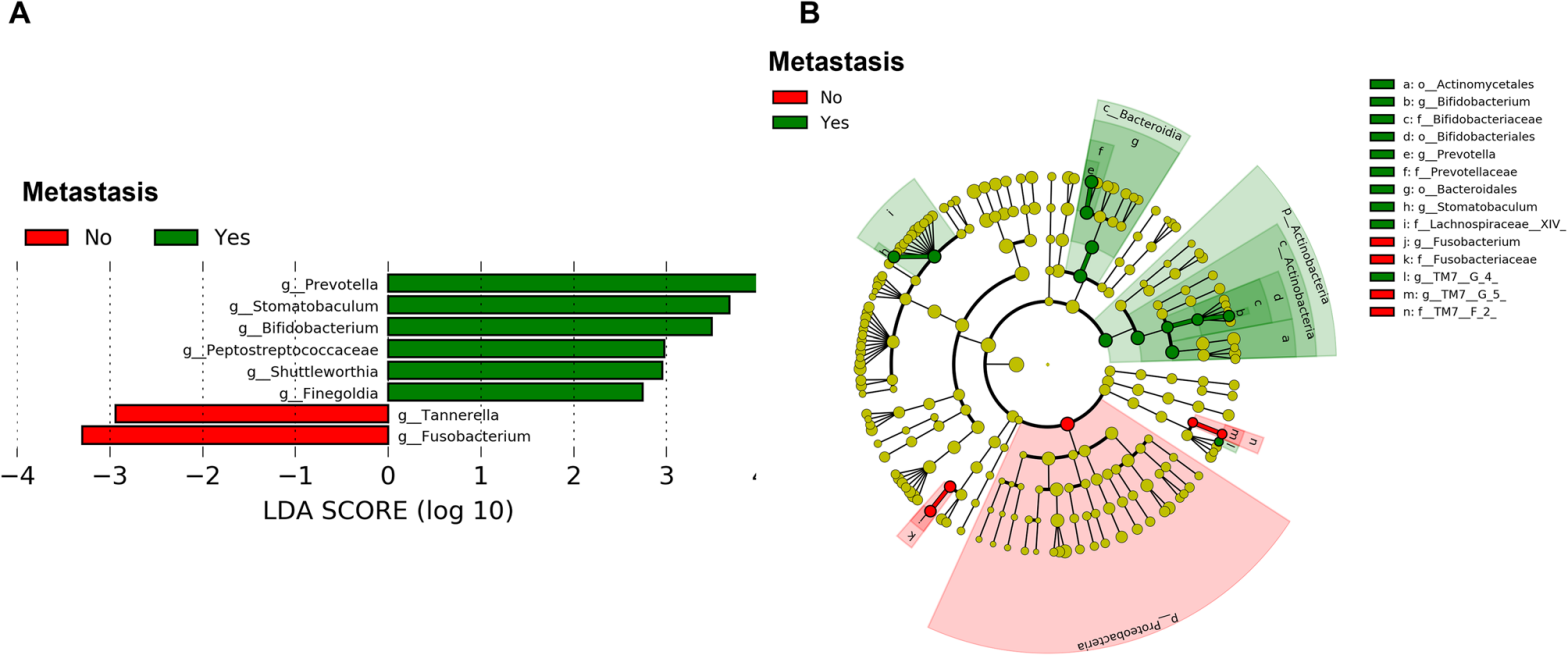
LDA effect size analysis showing differentially abundant taxa between patients with LNM and non-LNM with a threshold of |log10 LDA|≥ 2. Bar chart showing Log10 LDA of differential taxa in OSCC patients between metastasis and no metastasis groups (**A**). Cladogram showing the hierarchy among discriminative taxa. From phylum to genus, each node represents a taxonomic level. Red nodes demonstrate enriched taxa in the no metastasis group; green nodes represent enriched taxa in the metastasis group, LDA; linear discriminant analysis, OSCC; oral squamous cell carcinoma.

We performed LEfSe analysis at the genus levels, resulting in 30 differential features between patients in metastasis and no metastasis groups. The genera Prevotella, Stomatobaculum, Bifidobacterium, Peptostreptococcaceae, Shuttleworthia and Finegoldia were enriched in patients with metastasis. The genus Tannerella and Fusobacterium were dominant in patients without metastasis. A cladogram representing the differential phylogenic taxonomic classifications (Fig. 5A) with a threshold of |log10 LDA|≥ 2 is shown in Fig. 5B.

**Figure 6:**
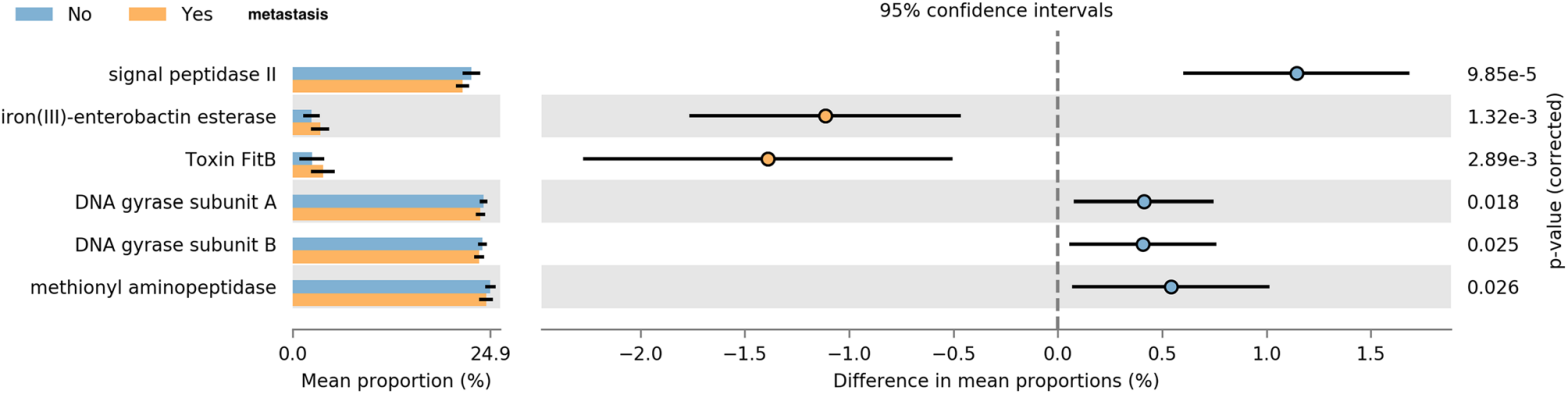
Prediction of altered KEGG pathways using PICRUSt2 analysis. A total of six KEGG pathways were significantly different between the metastasis and no metastasis groups. Bar plots on the left side display the mean proportion of each KEGG pathway. Dot plots on the right show the differences in mean proportions between the two indicated groups using P-values.

In addition, the functional diversity of the different putative metagenomes was assessed using PICRUSt2 software. In order to determine differences in the functional potential of the oral microbiota in OSCC patients, we predicted the metagenomes of the oral microbiota using the 16S rRNA gene dataset and compared differences in predicted functional gene abundance with Kyoto Encyclopedia of Genes and Genomes (KEGG) orthologs^17^. Pathways with a significant difference in mean proportions between the metastasis and no metastasis groups were identified. Pathways related to iron (III)-enterobactin esterase (*p* = 0.001) and toxin FitB (*p* = 0.002) were over-represented in the LNM group, whereas signal peptidase II (*p* < 0.001), DNA gyrase subunit A (*p* = 0.018), subunit B (*p* = 0.025), and methyonyl aminopeptidase (*p* = 0.026) were under-represented (Fig. 6).

### Microbial machine learning model for predicting lymph node metastasis

**Figure 7:**
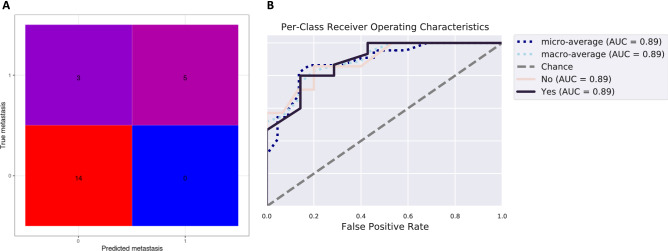
Evaluation of the prediction model for lymph node metastasis in oral squamous cell carcinoma. Confusion matrix (**A**) and the average receiver operating characteristic curves from the machine learning-based models (**B**).

The random forest classifier machine learning technique was used to predict whether a sample was from a patient with metastasis. The estimator parameter, which adjusts the number of trees grown by the ensemble estimators, was 200. The sample dataset was partitioned at 80:20 ratio, using 80% of the data to train the machine learning algorithms and the remaining 20% for the independent testing. We performed k-fold cross- validation (k = 5) during training of the machine learning algorithm. We presented classification accuracy results in the form of a confusion matrix, as well as receiver operating characteristic (ROC) curves. The confusion matrix shows that the overall accuracy of prediction is 86.3%, indicating a moderately high predictive accuracy (Fig. 7A). ROC analysis showing true and false positive rates for each group indicated good predictive accuracy for both metastasis (AUC = 0.89) and non-metastasis groups (AUC = 0.89) (Fig. 7B), indicating that the oral microbiome could be employed to predict LN metastasis in OSCC patients.

## Discussion

We compared the oral microbiome of saliva samples obtained before surgery from patients with or without LN metastasis. In the diversity analysis between metastasis and no metastasis groups, there was a statistically significant difference in the β-diversity, which represents the difference in the composition of the oral bacterial community in the two groups. Several genera were differentially identified in patients with metastasis. To our knowledge, this is the first study to identify an association between the oral microbiome and LN metastasis in OSCC.

Three theories have been proposed to explain the mechanisms of oral microflora in carcinogenesis. First, inflammatory mediators generated through chronic inflammation induced by bacteria can induce or promote cell proliferation, mutagenesis, oncogene activation, and angiogenesis^18^. Second, activation of NF-κB and inhibition of cell death by the oral microbiome can affect cell proliferation and cause carcinogenesis^19^. Finally, bacteria can produce metabolites that affect carcinogenic pathways^20^. Among the mechanisms that influence the tumor microenvironment, the main pathway of signaling between the microbiome and host is the production of bacterial metabolites. *Fusobacterium nucleatum* is known to be involved in the metastasis process and is predicted to activate β-catenin signaling through lipopolysaccharide (LPS). It has been reported that overexpression of β -catenin, and the oncogenes C-myc and cyclin D1, are involved in metastasis^21,22^. Miko et al. demonstrated that lithocholic acid (LCA), a secondary bile acid produced exclusively by bacteria, inhibits epithelial-mesenchymal transition (EMT) and vascular endothelial growth factor A (VEGFA) expression and boosts antitumor immunity in a breast cancer cell line^23^. However, there is a lack of research on the oral microbiome and its association with metastasis in oral cancer.

Therefore, we hypothesized that oral microbiota related to oral cancer metastasis could be found through differences in oral microbial communities in OSCC patients. We analyzed whether metastasis in OSCC could be predicted through the composition of the oral microbiome using a machine-learning model (RF). Modest predictive performance was achieved using taxonomy features. LEfSe analysis indicated that there were 30 features differentiating patients with LN metastasis from patients without it. The genera Prevotella, Stomatobaculum, Bifidobacterium, and Fusobacterium were the most prevalent differentiating features between with and without metastasis groups. Prevotella, a gram-negative bacteria associated with the pathogenesis of periodontitis, has been reported to be detected in OSCC^24,25^. In particular, *Prevotella inermedia* produces methyl mercaptan, which is involved in activation stress and DNA damage in oral cells. This methyl mercaptan is involved in angiogenesis and invasiveness of oral cancer cell through type 4 collagen cleavage^26^. Stomatobaculum is a genus of anaerobic bacteria of the Lachnospiraceae family, and *Stomatobaculum longum* belongs to this genus. *Stomatobaculum longum* is usually isolated from dental plaques^27^. Interestingly, in previous studies, Stomatobaculum were reported to be significantly abundant in oral swab samples of control patients compared to those with oral cancer^28^. However, according to our results, an increase in these taxa may be associated with metastasis in OSCC. Previous studies have suggested that the role of the oral microbiome in the aggressiveness of cancer in oral cancer is the induction of inflammation. It has been reported that infection with *Porphyromonas gingivalis* induces EMT by downregulation of E-cadherin and nuclear cytoplasmic accumulation of β-catenin, which induces aggressive and/or metastatic potential in OSCC^29^. However, in this study, no significant difference in abundance of *Porphyromonas gingivalis* was observed between the two groups. It has been demonstrated that Bifidobacterium can modulate host immunity and immune responses, alleviate allergic symptoms, treat inflammatory diseases, and have synergistic effects on tumor immunotherapy^30,31^. However, in this study, Bifidobacterium were significantly increased in the saliva of patients with metastatic oral cancer.

To characterize the functional role of the oral microbiota in metastasis, we annotated the taxa using the Kyoto Gene and Genome Encyclopedia (KEGG) database. The pathway that showed the most significant difference between the two groups was signal peptidase II. Signal peptidase II is present in bacterial inner membranes and releases signal peptides from murein prolipoproteins and other bacterial membrane proteins. It serves as a potential target for the development of antibacterial agents with a novel mode of action, as it cleaves off the signal peptide from secreted proteins, making it essential for protein secretion, and hence for bacterial viability^32^. Future studies are required to determine this enzyme's role in metastasis.

The strength of this study is that it evaluated potential oral microbiome biomarkers for the prediction of LNM in OSCC patients through various analyses. The depth of invasion (DOI) of OSCC is the most commonly used histopathologic variable for predicting occult nodal metastasis^33^. Other clinicopathologic factors have been reported to predict nodal disease, including sex, tumor location, histologic grade, pattern of invasion, and perineural and lymphovascular invasion^34^. In addition to the clinicopathological factors, this study showed that the composition of the oral microbiome could be another predictive factor for OSCC metastasis. While many existing studies have focused on the role of the microbiome in carcinogenesis of oral cancer, this is the first study to show that there is a change in the oral microbiome after metastasis. However, our study has some limitations. First, this study had a small sample size. Another limitation is that since this study investigated samples from patients of a single ethnicity and from a single geographical site, the results may not be generalizable to other groups, especially considering differences in the oral microbiome based on dietary habits. In addition, 16s rRNA amplicon sequencing may have limitations in identifying activated bacteria associated with metastasis in saliva. Amplicon sequencing can be applied to low-biomass specimens or samples contaminated by host DNA. Many species of oral bacteria are identical along the full length of the 16s rRNA gene, and in principle it therefore is impossible to distinguish all bacterial species using that gene. In general, 16s rRNA amplicon sequencing allows genus-level resolution for most bacterial taxa, but difficult species resolution^35^. Metatranscriptomics or whole-genome shotgun (WGS) sequencing analysis will provide more information for the determination of functionally relevant salivary microbiome. Finally, we did not consider sufficient information about oral health conditions that could affect the oral microbiome of the participants. Factors related to the wearing of the prosthesis were considered, but the influence of other oral health factors could not be excluded.

## Conclusion

We uncovered differentiated microbial communities and functions following LN metastasis in patients with OSCC undergoing surgery. It was confirmed that Prevotella, Stomatobaculum, Bifidobacterium were differential features of saliva microbiomes at the genus level in patients with LN metastasis. On the other hand, Fusobacterium were decreased in patients with LN metastasis. Additionally, LN metastasis can be predicted using machine-learning techniques based on oral microbiome features. However, based on the results of this preliminary study, mechanism study and large-scale sample analyses are required to validate the association between the oral microbiome and metastasis.

## Methods

### Sample collection

This study was approved by the Institutional Review Board of Kyung Hee University Hospital at Gangdong (IRB No: KHNMC 2018-06-023). All participants gave written, informed consent for their participation. Informed consent was obtained according to the Declaration of Helsinki. Between 2018 and 2019, 54 saliva samples were collected before surgery. Patients newly diagnosed with OSCC were enrolled in this study. All participants did not take any oral antibiotics at the time of saliva sample collection other than prophylactic intravenous antibiotics just before surgery. Briefly, OSCC patients were instructed to refrain from oral hygiene procedures such as brushing their teeth, eating, drinking, or smoking for a minimum of two hours prior to saliva sample collection. At least 1 mL of saliva was accumulated on the bottom of the oral cavity and then spit naturally into specimen tubes for collection. Each sample tube was immediately sealed and stored at − 80 °C until analysis.

### DNA extraction

Frozen saliva samples were thawed at room temperature for 1 h and subsequently incubated at 37 °C with shaking for 1 h after adding 1M dithiothreitol (Sigma-Aldrich, Chemie, Steinheim, Germany) to a final concentration of 65 mM, allowing bacterial cells to be released from the mucus portion of saliva samples^36^. After incubation, the samples were centrifuged at 8000 rpm to harvest pellets. Genomic DNA was extracted from the pellets using the DNeasy® PowerSoil Pro kit (Qiagen, Germany) according to the manufacturer’s instructions. Finally, the extracted DNA was eluted in autoclaved distilled water, and the yield was measured using a NanoDrop™ 2000 spectrophotometer (Thermo Scientific, USA).

### 16s rRNA amplicon sequencing

Genomic DNA from the saliva samples was used for profiling the oral microbial community through 16 s rRNA amplicon sequencing on the Illumina MiSeq platform. Primer combinations of 341 F (5-TCGTCGGCAGCGTC-AGATGTGTATAAGAGACAG-CCTACGGGNGGCWGCAG-3) and 805R (5-GTCTCGTGGGCTCGG-AGATGTGTATAAGAGACAG-GACTACHVGGGTATCTAATCC-3) targeting the V3-V4 hypervariable regions of the 16s rRNA gene were used to construct the 16s rRNA amplicon from the extracted DNA with Premix Taq™ (Ex Taq Version 2.0) (TaKaRa, Kyoto, Japan). Subsequently, the amplicons were purified using a QIAquick PCR Purification Kit (Qiagen). The purified amplicons were amplified with unique pairs of i5 forward index (5-AATGATACGGCGACCACCGAGATCTACAC-[i5]-TCGTCGGCAGCGTC-3) and i7 reverse index (5-CAAGCAGAAGACGGCATACGAGAT-[i7]-GTCTCGTGGGCTCGG-3) primers provided by the Nextera XT Index kit (Illumina, San Diego, CA, USA). Each set of PCRs included extraction and PCR blanks. Both [i5] and [i7] are the 8 bp sequences of unique barcodes, which allowed the pooled libraries to be sequenced simultaneously, known as sample multiplexing. The index barcoded amplicons were purified using a QIAquick PCR Purification kit (Qiagen, Germany) and quantified using a NanoDrop™ 2000 spectrophotometer (Thermo Scientific, USA). Equal amounts of each purified amplicon containing barcode were pooled to construct libraries. Libraries were sequenced on the Illumina MiSeq platform using the 2 × 300 bp paired-end protocol.

### Bioinformatic and statistical analysis

Raw sequence data were analyzed using the QIIME 2 pipeline (https://qiime2.org/) (version 2020.6) and phyloseq R package (https://joey711.github.io/phyloseq/)^37^. Raw sequence data were denoised using DADA2 in the QIIME 2 package^38^. The paired-end FASTQ files were processed by Phred quality score-based quality filtering, merging of the paired ends, chimera removal, singleton removal, and construction of a feature table consisting of amplicon sequence variants (ASV). Using DADA2 denoise-single method, we removed low quality regions of the sequences. We set the truncating sequence option to 260. All ASV were aligned to mafft through q2 alignment, and phylogenetic analysis was performed using fasttree2. A scikit­learn naïve Bayes machine­learning taxonomy classifier against the eHOMD(Human oral microbiome database, http://www.homd.org/) 16S rRNA RefSeq Version 15.1 reference sequences was trained with the q2­feature­classifier plugin using the V3­V4 regions of 16S rRNA sequences^39^. Taxonomy was assigned to each ASV using the ‘classifysklearn’ command in q2­feature­classifier. Using the constructed rooted phylogenic tree, we performed diversity analysis using the diversity plugin within the QIIME 2 package. The α-diversity and β-diversity were calculated between groups using the Kruskal–Wallis pairwise test and permutational multivariate analysis of variance, respectively. We analyzed differential microbial abundance between the two groups with Linear discriminant analysis Effect Size (LEfSe) in the Galaxy web application (http://huttenhower.sph.harvard.edu/galaxy)^40^. The alpha value for the factorial Kruskal–Wallis test among classes was 0.5, and the threshold on the logarithmic LDA score for discriminative features was 3.0. The strategy for multiclass analysis was all-against-all, which is the relatively stricter option. Based on the LEfSe results, with a threshold of |log10 LDA|≥ 2, a cladogram was plotted. Functional metagenomes were predicted based on the 16S rRNA sequencing data of the salivary microbiome using PICRUSt2 (phylogenetic investigation of communities by reconstruction of unobserved states) v2.0.3 (https://github.com/picrust/picrust2)^41^. We also performed KO (KEGG Orthology) pathway prediction using metagenome data.

## Supplementary Information

Supplementary Information 1.

Supplementary Information 2.

Supplementary Information 3.

Supplementary Information 4.

Supplementary Information 5.
