# Supplementary material for: Oral microbiome associated with lymph node metastasis in oral squamous cell carcinoma
Source: Sci Rep. 2021 Nov 30;11:23176. doi: 10.1038/s41598-021-02638-9 (PMC8633319; doi:10.1038/s41598-021-02638-9)
Supplement: Supplementary file 4 — Supplementary Information 4. [file 41598_2021_2638_MOESM4_ESM.docx]

Supplemental Figure 1. Oral bacterial communities were explored at the genus level in patients.
