# Supplementary figures and images for: Oral microbiome associated with lymph node metastasis in oral squamous cell carcinoma
Source: Sci Rep. 2021 Nov 30;11:23176. doi: 10.1038/s41598-021-02638-9 (PMC8633319; doi:10.1038/s41598-021-02638-9)

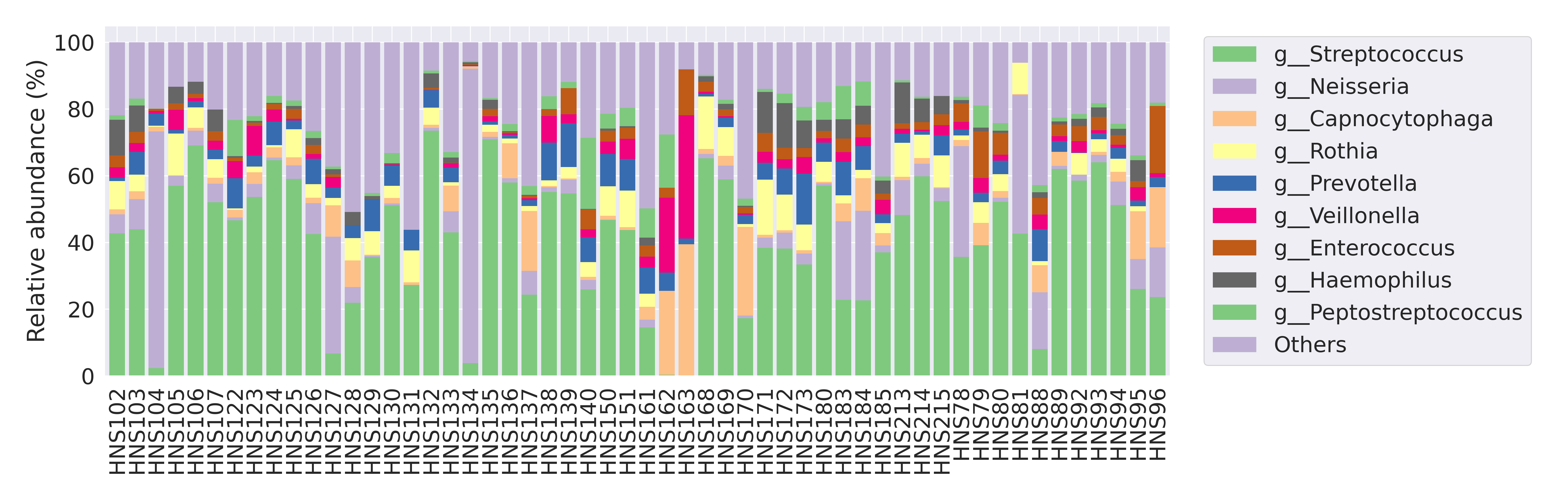

Supplement: Supplementary file 5 — Supplementary Information 5. [file 41598_2021_2638_MOESM5_ESM.png]
